# Diagnosis and management of Alagille and progressive familial intrahepatic cholestasis

**DOI:** 10.1097/HC9.0000000000000314

**Published:** 2023-12-07

**Authors:** Katherine Cheng, Philip Rosenthal

**Affiliations:** 1 Department of Pediatrics Gastroenterology, Hepatology and Nutrition, University of California San Francisco, San Francisco, California, USA; 2 Department of Surgery, University of California, San Francisco, San Francisco, California, USA

## Abstract

Alagille syndrome and progressive familial intrahepatic cholestasis are conditions that can affect multiple organs. Advancements in molecular testing have aided in the diagnosis of both. The impairment of normal bile flow and secretion leads to the various hepatic manifestations of these diseases. Medical management of Alagille syndrome and progressive familial intrahepatic cholestasis remains mostly targeted on supportive care focusing on quality of life, cholestasis, and fat-soluble vitamin deficiency. The most difficult therapeutic issue is typically related to pruritus, which can be managed by various medications such as ursodeoxycholic acid, rifampin, cholestyramine, and antihistamines. Surgical operations were previously used to disrupt enterohepatic recirculation, but recent medical advancements in the use of ileal bile acid transport inhibitors have shown great efficacy for the treatment of pruritus in both Alagille syndrome and progressive familial intrahepatic cholestasis.

## INTRODUCTION

Alagille syndrome and progressive familial intrahepatic cholestasis (PFIC) are conditions that can affect multiple organs, as well as lead to the need for a liver transplant. The impairment of normal bile flow and secretion leads to the various hepatic manifestations of these diseases. Bile is an alkaline fluid that is produced by hepatocytes from bilirubin, bile acids, and various lipids including cholesterol. Bile acid secretion is a complex process. The integrity of the hepatocyte is dependent on tight junction protein (TJP2) that protects hepatocytes from the detergent properties of bile. The bile salt export pump (BSEP) on the canicular membrane, which is controlled by the farnesoid X receptor, transports the newly synthesized bile acids. Localization of the apical membrane, such as BSEP, is mediated by myosin 5B (MYO5B). Phospholipid and cholesterol are secreted into the bile duct by means of the multi-drug resistance–associated protein 3 (MDR3) and familial intrahepatic cholestasis 1 (FIC1) protein. Bile is then transported to and stored in the gallbladder, where it is then released into the small intestine to aid lipid digestion and fat-soluble vitamin absorption.^1^ Once excreted into the gut, some of the bile acids are then reabsorbed into the ileum back to the liver through the enterohepatic circulation using the apical sodium-dependent bile transporter.

### Alagille syndrome

Alagille syndrome is an autosomal dominant disease with variable phenotypic penetrance that was first described in 1969 by Daniel Alagille as a constellation of clinical features in various organ systems.^2^

It is caused by various mutations in jagged canonical Notch ligand 1 (*JAG1*), which is a ligand in the Notch signaling pathway.^3,4^ Notch signaling is essential for normal embryonic development.^5^ Most patients have a detectable mutation in the *JAG1* gene (estimated 94%), and a small percentage with a mutation in *NOTCH2*.^6,7^ The same genetic mutation can have different phenotypic characteristics within the same family and there is still ongoing study into genetic modifiers for this disease.

Alagille syndrome was previously estimated to have frequency of 1 in 70,000 live births; however, with advances in molecular diagnosis, the true frequency is thought to be closer to 1 in 30,000 as many individuals do not have neonatal liver disease.^8^

### Multi-organ involvement of Alagille syndrome

#### Hepatic involvement

While the initial description of Alagille syndrome was based on bile duct paucity on liver biopsy, the more recent report found that hepatic involvement is not always present for children and adults with Alagille syndrome.^2^ A case series from King’s College reports only 75% of patients with Alagille syndrome had bile duct paucity, and 89% with cholestasis.^9^ Cholestasis can also vary from mild to severe, and synthetic dysfunction is typically rare. Serum aminotransferases can be elevated but may also be normal in some with cholestasis. When coagulopathy is present, it is often due to vitamin K deficiency. Those with hepatic involvement typically present in the neonatal period with direct or conjugated hyperbilirubinemia, and those with mild disease often show improvement by early childhood. There is typically no late-onset cholestasis outside of childhood.

Bile duct paucity is the most consistent feature of Alagille syndrome. While the normal bile duct to portal space ratio is between 0.9 and 1.8, bile duct paucity is defined as a ratio of <0.9 in full-term or older infants.^2^ Many pathologists often require at least 10 portal tracts to evaluate for bile duct paucity.^10^ In a study of 81 children with Alagille syndrome who underwent liver biopsy, 85% had bile duct paucity, but it was present in only 60% of liver biopsies done prior to 6 months of age compared to 95% of those done after 6 months of age.^11^ Ductular proliferation and giant cell hepatitis can also be seen in some infants and can lead to a misdiagnosis of biliary atresia. The progression of bile duct paucity during infancy is unclear but likely related to bile duct development postnatally.^2^

Pruritus often develops early and is disproportionately more severe than the degree of hyperbilirubinemia. It rarely is present before 3–5 months of age but is seen in most children with Alagille syndrome by 3 years. This often can be debilitating. Serum bile acid levels can be elevated even in the case of normal serum bilirubin. Patients with impaired bile secretion can have reduced secretion of cholesterol with cholesterol levels >1000 mg/dL, often resulting in formation of xanthomas, which develop over the first few years of life and resolve as cholestasis improves. Xanthomas typically form on the extensor surfaces of fingers, the palmar creases, napes of neck, the ears, popliteal fossa, buttocks, and around the inguinal creases. Of note, the type of hyperlipidemia from Alagille syndrome is predominately lipoprotein X and not atherogenic.

Children with significant cholestasis during infancy may have a more severe clinical course in the first 5 years of life, after which it can improve in many children. This spontaneous improvement is poorly understood but well documented. It is difficult to predict whether those with cholestasis will have improvement or progression of their liver disease overtime. Kamath et al^12^ showed in a small cohort of children with Alagille syndrome that total bilirubin >6.5 mg/dL, conjugated bilirubin >4.5 mg/dL, and cholesterol >520 mg/dL under age 5 are associated with more severe liver disease, defined as severe cholestasis with pruritus, cirrhosis, portal hypertension, or need for biliary diversion or liver transplant. The Global Alagille Alliance (GALA) study group was formed as an international consortium to better elucidate the natural history of liver disease in children with Alagille syndrome.^13^ This study found that compared to children with median total bilirubin <5 mg/dL, those with bilirubin between 5 and 10 mg/dL had an almost 5-fold increased risk of liver transplant, and 4.1-fold increased risk of portal hypertension.^13^ Children with serum bilirubin >10 mg/dL had a 15.6-fold increased risk for liver transplant and an 8-fold increased risk for portal hypertension.^13^

HCC is rare but can occur even in very young children with Alagille syndrome, with or without cirrhosis.^13,14^ Patients with Alagille syndrome are more likely to develop regenerative nodules, and this can be solitary and adjacent to the right portal vein.^15^

#### Extrahepatic involvement

Cardiac disease is a common feature of Alagille syndrome and found in up to 94% of patients. Pulmonary artery anomalies are the most common feature, occurring in 76% of those with cardiac involvement.^16^ The most common congenital defect is tetralogy of Fallot, which occurs in 7%–12%.^11,16^ Cardiac involvement is associated with increased mortality and accounts for nearly all of the early deaths in Alagille syndrome with 40% 6-year survival compared to 95% in those without intracardiac disease.^11^

While pulmonary artery involvement is a hallmark feature of Alagille syndrome, there are widespread vascular anomalies that can be seen in Alagille syndrome, often thought to be due to the intrinsic role of the Notch signaling pathway in vascular morphogenesis, angiogenesis, and vessel homeostasis.^17^ Intracranial bleeds occur in 22%–25% of patients; 30%–50% of these events are fatal even without significant coagulopathy.^11,18,19^ Systemic vascular anomalies involving the aorta, renal, celiac, superior mesenteric, and subclavian arteries have also been reported.^20^ Screening MRI studies of the brain will detect various vascular abnormalities in >30% of patients, many of whom are asymptomatic.^21,22^ Given the relatively high prevalence of silent vascular disease, it is currently recommended that all children with Alagille syndrome have a screening MRI or magnetic resonance angiogram when they reach an age when they no longer require general anesthesia for undergoing an MRI. Clinicians should also have a low threshold to repeat imaging in case of trauma or neurologic symptoms. There are currently no Alagille-specific recommendations for the treatment of cerebrovascular anomalies, although referral to a neurosurgeon is typically warranted.

There are a wide range of vertebral anomalies described, but the most characteristic skeletal abnormalities seen in Alagille syndrome remain the sagittal cleft, or butterfly vertebrae. This occurs when the anterior arches of the vertebral bodies fail to fuse, and the vertebrae are split sagittally into hemivertebrae.^2^ Patients are asymptomatic but this can occur in 33%–66% of patients with Alagille syndrome with the large range of frequency likely due to discrepancies in detection.^11,23,24^ Children with Alagille syndrome have an increased fracture risk, especially femur fractures even in the presence of little to no trauma.^25^ This is likely multifactorial from cholestasis, malabsorption, fat-soluble vitamin deficiencies as well as chronic hepatic and renal disease. Using data from The Childhood Liver Disease Research Network, Loomes et al found that among those with Alagille syndrome, Dual-energy x-ray Absorptiometry Z-scores were negatively correlated with serum total bilirubin and bile acid levels.

There is a wide range of prevalence of renal involvement in patients with Alagille syndrome, reporting up to 70%.^13,27^ The most common renal involvement is renal dysplasia (58.9%, followed by renal tubular acidosis (9.5%).^28^ Despite renal involvement, end-stage renal disease requiring renal replacement therapy or transplant is uncommon; however, functional and structural evaluation of the kidneys should be done in all children and adults with Alagille syndrome, especially those undergoing liver transplant evaluation. Using data from the Society of Pediatric Liver Transplantation network, Kamath et al^29^ found that patients with Alagille syndrome were more likely to have renal impairment before liver transplant compared to those with biliary atresia with calculated glomerular filtration rate of <90 mL/min/1.73 m^2^ in 18% of patients with Alagille syndrome compared to 5% of those with biliary atresia (*p*<0.001). Additionally, children with Alagille syndrome had higher serum creatinine than children with biliary atresia before transplant. Two years after liver transplant, renal insufficiency is present in 22% of cohort with Alagille syndrome, compared to 8% of patients with biliary atresia, suggesting that careful monitoring and potentially renal-sparing immunosuppressive protocols should be considered.

The ocular abnormalities seen in patients with Alagille syndrome do not typically affect vision and include a wide range of findings including posterior embryotoxon, iris abnormalities, diffuse fundic hypopigmentation, and optic disc abnormalities. Posterior embryotoxon is the most common finding and present in 56%–95% of those with Alagille syndrome.^30,31^ It is a prominent, centrally positioned ring or line at the Schwalbe ring where the corneal endothelium and uveal trabecular meshwork join. The high frequency of ocular findings suggests that formal ophthalmologic slit-lamp examination can be helpful for diagnostic purposes. Of note, in one study, 22% of children evaluated in the general ophthalmology clinic had posterior embryotoxon.^30^ Nischal et al^32^ found that ultrasound evidence of optic disk drusen in at least 1 eye was present in 95%, and bilateral disc drusen in 80% of those with Alagille syndrome, compared to none in those without Alagille syndrome, suggesting this may be useful for diagnosis.

The typical patient with Alagille syndrome is described as having triangular facies with a prominent forehead, deep-set eyes with hypertelorism, saddle or straight nose, and pointed chin. While this can be present during infancy, it generally becomes more pronounced with age.

Children with Alagille syndrome are known to be smaller in height and weight compared to their peers, with more than half falling below the fifth percentile for height and/or weight.^33^ The growth impairment is thought to be multifactorial, related to cholestasis, malabsorption, and chronic liver and kidney disease. Diarrhea is common for children with Alagille syndrome and was initially thought to be related to pancreatic insufficiency; however, the most recent study showed no difference in fecal elastase in children with Alagille syndrome and routine screening for pancreatic insufficiency is not currently recommended.^34^

Due to the multi-organ involvement of Alagille syndrome, assessment of neurocognitive outcomes and health-related quality of life is complex. The cholestasis-related pruritus is typically the most severe of all liver diseases, often resulting in cutaneous marks, as well as affecting sleep and school. In a large cohort study using the Pediatric Quality of Life Inventory (PedsQL) Generic Core scale, children with Alagille syndrome were found to have lower self-reported and parent-reported PedsQL 4.0 scores compared to matched healthy controls and those with alpha-1 antitrypsin deficiency, with the largest discrepancy seen in physical domain.^35^

### Diagnosis

The majority of infants with Alagille syndrome are evaluated for conjugated hyperbilirubinemia within the first few months of life, and this should include blood work, and imaging (often ultrasound to start). The diagnosis can be made based on a liver biopsy showing intrahepatic bile duct paucity, along with at least three of the major clinical features: chronic cholestasis, cardiac disease, skeletal abnormalities, ocular abnormalities, or characteristic facial features. While not part of the diagnostic criteria, there is also a high prevalence of renal and vascular disease in these patients.^11,20^ In the era of molecular testing, this has been very helpful for confirming a diagnosis.

### Progressive familial intrahepatic cholestasis

PFIC refers to a heterogenous group of monogenic disorders that result from defects in canalicular bile acid trafficking and/or secretion.^1^ Any interruption or disruption in the process can result in intermittent cholestatic jaundice such as benign recurrent intrahepatic cholestasis (BRIC), or chronic liver disease (PFIC). The rarity of this disease makes it difficult to study, but multicenter collaborations such as the ChiLDREN network and the Natural Course and Prognosis of PFIC and Effect of Biliary Diversion have been extremely helpful in providing more insights into this group of diseases.

PFIC disorders are autosomal recessive but those with a heterozygous mutation may present with neonatal cholestasis, intrahepatic cholestasis of pregnancy, cholelithiasis or choledocholithiasis, predisposition to DILI or parenteral nutrition-associated liver disease.^36–38^ Pruritus remains the most significant clinical manifestation of these disorders and can affect school, sleep, and overall quality of life.

### Specific PFIC diseases (Table 1)

#### PFIC1 [ATPase phospholipid transporting 8B1 gene deficiency]

PFIC1 (also known as Byler disease) and BRIC are both caused by a mutation in the ATPase phospholipid transporting 8B1 (*ATP8B1*) gene, located on chromosome 18, which encodes for *FIC1*.^39^ This leads to membrane instability and inability to traffic bile acids. Patients with PFIC1 typically present with cholestasis that starts in infancy and progresses to cirrhosis, typically by the second decade of life.^40^ Serum gamma-glutamyl transferase (GGT) is typically normal with minimally elevated serum aminotransferases. Liver biopsy usually reveals bland cholestasis and granular “Byler” bile on electron microscopy.^41^ Diarrhea, malabsorption, and failure to thrive are common in early childhood. Fat-soluble vitamin malabsorption can lead to catastrophic bleeding (vitamin K deficiency), rickets (vitamin D deficiency), and neuromuscular dysfunction (vitamin E deficiency). Despite its early onset of cholestasis and jaundice, pruritus may not be noted until six months of age due to a lack of fully developed neural pathways for itching, but affected infants are often irritable.^42^ Unlike other cholestatic disorders, these patients do not develop xanthomas. Without treatment, these children often have growth failure with short stature, delayed onset of puberty, and sexual development. FIC1 is also expressed in the pancreas, ears, small intestine, and bladder, which leads to patients having extrahepatic manifestations such as recurrent pancreatitis, diarrhea, and sensorineural hearing loss. These extrahepatic manifestations often persist and can even worsen after a liver transplant.^43^ Severe allograft steatosis can occur after a liver transplant and can be progressive leading to cirrhosis (Table 1).^44^

**TABLE 1:** Different types of PFIC

Disease	Chromosome	Gene	Protein	Significant lab findings	Other findings
Type 1	18q21-22	ATP8B1	Familial intrahepatic cholestasis (FIC1)	Low or normal GGT	Diarrhea, growth failure, hearing loss, pancreatitis, respiratory disease
Type 2	2q24	ABCB11	Bile salt export pump (BSEP)	Low or normal GGT elevated AFP	HCC
Type 3	7q21	ABCB4	Multi-drug resistance–associated protein 3 (MDR3)	Elevated GGT	Cholesterol cholelithiasis
Type 4	9q21	TJP2	Tight junction protein 2 (TJP2)	Low or normal GGT	Respiratory disease, hearing loss, neurologic symptoms
Type 5	12q23	NR1H4	Farnesoid X receptor (FXR)	Low GGT Elevated AFP	Rapidly progressive cholestasis, coagulopathy
Type 6	18q21.1	MYO5B	Myosin 5B	Low GGT	Hepatomegaly

Abbreviations: *ABCB4*, ATP-binding cassette subfamily B member 4; *ABCB11*, ATP-binding cassette subfamily B member11; *ATP8B1*, ATPase phospholipid transporting 8B1; GGT, gamma-glutamyl transferase; *MYO5B*, myosin 5B; *NR1H4*, nuclear receptor subfamily 1, group H, member 4; PFIC, progressive familial intrahepatic cholestasis; *TJP2*, tight junction protein 2.

#### PFIC2 (ATP-binding cassette subfamily B member11 deficiency)

PFIC2 is caused by a mutation in ATP-binding cassette subfamily B member11 (*ABCB11*) located on chromosome 2, which encodes for *BSEP.* This is the most common form of PFIC and typically presents in the neonatal period with progressive cholestasis.^40^ There is a clear association between mutation severity and disease as those with partial loss of BSEP function often have less severe disease, as well as improved native liver survival.^45^ Jansen et al^46^ found that among 7 patients treated with ursodeoxycholic acid, some were able to excrete very low amounts of ursodeoxycholic acid into bile, suggesting those patients with some residual BSEP function may respond to ursodeoxycholic acid. Without treatment, these patients have rapid progression to cirrhosis. Liver biopsy may show giant cell hepatitis, as well as cholestasis with amorphous bile and absence of BESP protein.^1^ Like PFIC1, these patients can have failure to thrive and soluble vitamin deficiencies, along with its associated complications. Hepatosplenomegaly can present due to portal hypertension that is related to advanced fibrosis or cirrhosis.^47^ Cholelithiasis is observed in 30% of patients, likely due to impaired bile acid secretion and supersaturation of bile with cholesterol. Like PFIC1, these patients do not have xanthomas. The lack of extrahepatic manifestations typically helps distinguish PFIC1 from PFIC2 as BSEP protein is expressed exclusively in the liver; however, patients with PFIC2 are at risk of developing HCC and cholangiocarcinoma and this can occur as early as 10 months of age.^40,48,49^ Like those with PFIC1, patients with PFIC2 also have low serum GGT, and normal or near-normal serum cholesterol levels.^40^ Unlike those with PFIC1, these patients typically have elevated serum aminotransferase at least 5 times the normal value.

Although liver transplant can be curative in PFIC2, anti-BSEP antibodies can develop and lead to recurrent liver disease after liver transplant.^50^ This can be treated with B-cell–depleting antibody therapy such as rituximab, intravenous immunoglobulin, plasmapheresis, and steroids.^51,52^

#### PFIC type 3 (ATP-binding cassette subfamily B member 4 [*ABCB4*] deficiency)

PFIC3, caused by mutation in ATP-binding cassette subfamily B member 4 (*ABCB4*) on chromosome 7, leads to MDR3 disease. It is distinguished from PFIC1 and PFIC2 by elevation in serum GGT.^40^ Due to the impaired neutralization of free bile acids that occur from mutations in the *ABCB4* gene, this causes low phospholipid bile and injury.^1^ Unlike the previous PFIC disorders, the age of symptom onset is broad ranging from infancy to early adulthood.^53^ A subgroup of patients can also develop intrahepatic cholestasis of pregnancy, DILI, or chronic cholangiopathy.^54^ Jacquemin et al^53^ described that among 31 patients with PFIC3, cholestasis can present in one-third of patients by 1 year with less severe pruritus compared to other PFIC. Splenomegaly is present in 87% of children at mean age of 5 years and 61% had esophageal varices with mean age of 9 years. Due to portal hypertension, liver failure or severe cholestasis, 58% of children required liver transplant at a mean age of 7.5 years. Liver biopsy in these patients may show bile duct proliferation and intraductal stone formation.^55^

Because *ABCB4* mutation can lead to low phospholipid-associated cholelithiasis syndrome, some patients can have cholesterol cholelithiasis typically before 40.^56^ Unfortunately, even after cholecystectomy, patients may still have recurrence of symptoms.

### Newer PFICs

#### PFIC4 (tight junction protein 2)

PFIC4 was first described in 2014. It is caused by loss of function mutation in *TJP2* gene on chromosome 9 that leads to TJP2 protein deficiency. This causes bile leakage between cells within the hepatic parenchyma, and damage to surrounding hepatocytes and cholangiocytes.^57^ Similar to PFIC2, the severity of disease varies depending on the mutation and penetrance.^58^ Complete TJP2 deficiency typically presents with severe progressive cholestasis starting in early childhood, with low GGT. PFIC4 has been associated with extrahepatic manifestations including respiratory disease, hearing loss, and neurologic symptoms due to widespread expression of TJP2.^59^ There have been reports of HCC in those with TJP2 deficiency^1,60^

#### PFIC5 (farnesoid X receptor disease)

PFIC5 is due to mutation in the nuclear receptor subfamily 1, group H, member 4 (*NR1H4*) gene on chromosome 12. This is responsible for imbalance in bile acid homeostasis, and inappropriate regulation of BSEP.^57^ This can lead to severe neonatal cholestasis, often before 2 months of age, along with coagulopathy that is refractory to vitamin K supplementation and rapid progression to end-stage liver disease. Serum GGT may be low, but another distinction is alpha-fetoprotein is often markedly elevated.^1^ Due to its rapid progression, diagnosis may not be made prior to a liver transplant.

#### PFIC6 (*MYO5B* disease)

PFIC6 is due to a mutation in the *MYO5B* gene on chromosome 18, which encodes for MYO5B protein. This leads to inappropriate cell membrane trafficking and location of BSEP and other proteins. While MYO5B has been associated with intestinal microvillous inclusion disorder, isolated cholestatic liver disease can also occur.^1^ These patients typically present in the first 2 years of life with low GGT cholestasis, mildly elevated serum aminotransferase, pruritus, and hepatomegaly.^57^

### BRIC

BRIC can be seen with defects in FIC1 and BSEP. These patients have attacks of jaundice and pruritus separated by symptom-free intervals.^42^ During attacks, patients may also have fatigue, anorexia, steatorrhea, dark-colored urine, and weight loss. Attacks can be preceded by minor illness, hormonal factors (eg, use of oral contraceptives or pregnancy). Those with *ATP8B1* gene mutation, can also have severe coughing during the episodes.

### Medical management of Alagille syndrome and PFIC

Medical management of Alagille syndrome and PFIC remains mostly targeted on supportive care focusing on quality of life, cholestasis, and fat-soluble vitamin deficiency (Table 2).

**TABLE 2:** Supportive care for children with Alagille syndrome and PFIC

	Medication/Treatment
Nutrition	MCT formula and supplement
	Consider nasogastric feedings
	Fat-soluble vitamin supplement
Antipruritic	Ursodeoxycholic acid
	Rifampin
	Cholestyramine
	Naltrexone
	Serotonin uptake inhibitor (eg, sertraline)
	Antihistamine
	Ileal bile acid transport inhibitors
Development	Neurodevelopment and monitoring (vascular imaging for children with Alagille syndrome)

Abbreviation: MCT, medium chain triglyceride; PFIC, progressive familial intrahepatic cholestasis.

Failure to thrive and malnutrition are common for children with Alagille syndrome and PFIC and require close monitoring and supplementation, especially with formula containing medium-chain triglycerides. Many also require vitamin supplements, especially fat-soluble vitamins (A, D, E, and K). Some patients may require nasogastric feeds or gastrostomy tube feedings to reach caloric intake goals.

The most difficult therapeutic issue remains the management of pruritus. Pruritis can have a significant impact on the quality of life for children and adults with these diseases. Skin emollients, trimmed fingernails, and avoiding hot baths can help minimize itching and excoriation. There are no specific guidelines to treat pruritus for these children, and medications are typically added in stepwise fashion to help control symptoms.

For children with Alagille syndrome, ursodeoxycholic acid, a choleretic that stimulates bile flow, is typically the first-line therapy to add and has been shown to be effective in treating some patients for pruritus and reducing xanthoma formation.^61^ Unfortunately, most children will require additional therapy to treat pruritus. Bile acid–binding resins such as cholestyramine may be helpful, but this can affect other medication absorption and must be given separately for at least 2 hours. It also increases the risk of fat-soluble vitamin deficiencies and needs to be monitored closely. Rifampin has been shown to be useful to treat pruritus when others have failed.^62^ To control mild itchiness or symptoms that interfere with sleep at night, antihistamines such as hydroxyzine or diphenhydramine can be used but these are typically not effective as monotherapy and effects are transient.^62^ Naltrexone, an opioid antagonist, has been found effective in pediatric cholestatic disease but there is currently no study on its use specifically for those with Alagille syndrome.^63^ Selective serotonin receptor inhibitors have been shown to be effective to treat pruritus in adults with cholestasis and there is limited data supporting the use of sertraline in children.^64^

Similarly, antihistamine and ursodeoxycholic acid can be trialed but are typically not very efficacious alone for children with PFIC. Ursodeoxycholic acid at daily dosage of 10–20 mg/kg may improve liver biochemical markers, but has little benefit in those with severe pruritus and PFIC1 or PFIC2.^65^ In those with PFIC3, ursodeoxycholic acid has been noted to lead to normalization of liver tests in half of the patients.^66^ Like patients with Alagille syndrome, other medications to consider include rifampin, cholestyramine, or antihistamines.

While surgical operations were thought to be the only option to disrupt the enterohepatic circulation, recent medical advancements in the use of ileal bile acid transport inhibitors have shown efficacy for the treatment of refractory pruritus in Alagille syndrome and PFIC. The apical sodium-dependent bile acid transporter is essential for reabsorption of bile acid in the ileum and enterohepatic recirculation.^67^ The inhibition of these transporters leads to increased bile acid load in the colon. And subsequently lower bile acid pool. There are 2 medications currently available, maralixibat and odevixibat.

Maralixibat is currently Food and Drug Administration approved for children 3 months and older with Alagille syndrome with cholestatic pruritus. ICONIC was the first international, multicenter, phase 2b, placebo-controlled, double-blind trial reviewing the efficacy and safety of maralixibat for children with Alagille syndrome.^68^ In this study, 31 children were enrolled and started on maralixibat for 18 weeks and then randomized to either remain on maralixibat or placebo from week 19 to 22, followed by open-label maralixibat at 380 µg/kg daily until week 48, with an optional long-term extension period thereafter. In this study, those who were randomized to placebo at week 19 had a significant increase in serum bile acid levels and pruritus while those who remained on maralixibat maintained treatment effect throughout the whole study period. Clinically, meaningful improvement in pruritus was achieved by week 3 of treatment with 43% of participants having minimal to no itch at week 48, and 93% at week 204. Participants with xanthoma at baseline also improved throughout the study period with no new xanthoma reported in those who received maxalixibat. There was a significant reduction in total cholesterol level from baseline to weeks 18 and 48. Participant’s quality of life was improved drastically from baseline to weeks 18 and 48. This study showed that maralixibat was generally well tolerated with the most frequent adverse events related to gastrointestinal side effects but were self-limited in nature. During this study, fat-soluble vitamin supplements were provided as standard of care.

INDIGO was an open-label, phase 2 international study on the efficacy and safety of maralixibat in children 1–18 years old with PFIC1 and PFIC2.^69^ Thirty-three children received daily maralixibat 266 μg/kg for 72 weeks and then up to twice daily dosing starting at 72 weeks up to 240 weeks. This study found a variable response depending on PFIC subtype, as well as BSEP mutation. Those with BSEP deficiency, especially those with residual BSEP function, experienced significant and sustained improvement in pruritis, biochemical laboratory parameters, growth, and quality of life. A phase 3 study is currently ongoing.

Odevixibat is another ileal bile acid transporter inhibitor. It is currently approved for pruritus in children with Alagille syndrome (12 mo and older) and all types of PFIC (3 mo and older).^70^ In a multicenter phase 2 open-label study, 20 children 1–17 years with various cholestatic diseases, including 6 with Alagille syndrome, were enrolled.^71^ In this study, participants received daily odevixibat for 4 weeks. Among those with Alagille syndrome, 5 of the 6 patients had reductions in serum bile acids, and most had improvement in pruritus and sleep scores. Like the maralixibat study, most of the adverse events were mild and transient. Odevixibat recently completed its phase 3 study, ASSERT trial, which is a double-blind, randomized, placebo-controlled trial on the use of odevixibat at 120 µg/kg/d for 24 weeks in children with Alagille syndrome.^72^ Like the ICONIC trial, odevixibat was associated with a significant reduction in pruritus compared to placebo, as well as a decrease in serum bile acid levels and sleep disturbance. In the phase 3, randomized, double-blind PEDFIC study, children with PFIC1 or PFIC2 were assigned to either placebo, odevixibat 40 μg/kg, or odevixibat 120 μg/kg over 24-week period.^73^ Treatment with odevixibat led to improvement in pruritus observed by week 4, and sustained improvement. Those who received odevixibat also had reduction in serum aminotransferase levels, as well as improved growth and sleep parameters. There were no serious adverse events reported, and medication was generally well tolerated for both doses of odevixibat. The recommended starting dose for odevixibat is 40 µg/kg. If no improvement in pruritis is noted after 3 months, the dosage can be increased in 40 µg/kg increments up to 120 µg/kg once daily.

Due to the risk of HCC in those with PFIC2, it is important to consider screening for hepatic malignancy with tumor markers (including alpha-fetoprotein) and imaging.

### Surgical management

For children and adults with Alagille syndrome or PFIC who have pruritus refractory to medical management, surgical intervention can be considered to aim to disrupt the enterohepatic circulation of bile acids to prevent intestinal re-uptake of bile acids and therefore decrease total body bile acids and allow pruritus to improve. Whitington and Whitington^74^ first described the use of partial external biliary diversion for children with intractable pruritus from cholestasis by using a jejunal conduit to drain the gallbladder externally and allow 30%–70% of bile to drain out of an ostomy and discarded.

In a multicenter study, partial external biliary diversion has been shown to be able to decrease patient-reported itch score and xanthoma in children with Alagille syndrome.^75^ Unfortunately, this is less effective for those with Alagille syndrome compared to PFIC as the biliary tree may be hypoplastic with less bile reaching the duodenum.^17^ For children and adults with PFIC, after partial external biliary diversion, most patients subjectively experience a response with one study reporting 81% of those with PFIC1 and 76% of those with PFIC2 having transient or sustained improvement of pruritus.^76^ A new variant of this procedure consists of an internal diversion to the colon to obviate the need for an ostomy bag. Internal partial ileal exclusion is less commonly used and involves transecting the distal 15% of small intestine and sewn end to side to the cecum, therefore allowing internal bypass of the distal ileum.^77^ There is limited data and experience with this surgical approach. Given the rarity of PFIC as well as the limited literature, Davis et al^78^ found in a review that there is no evidence of superiority for one type of nontransplant surgical intervention compared to another.

Hepatoportoenterostomy, a surgical operation commonly done for biliary atresia, is not indicated in Alagille syndrome or PFIC and can increase the amount of liver injury. One study found that among children with Alagille syndrome who underwent hepatoportoenterostomy, a higher percentage required liver transplantation (47% vs. 14%).^79^

### Despite medical management, some patients with Alagille syndrome or PFIC may still require a liver transplant

In the GALA study, the cumulative incidence of liver transplant at 5, 10, and 18 years was 27.1%, 37.8%, and 50.4%, with the median age of transplant at 2.8 years; 4 of the 1184 patients required combined liver-kidney transplant.^13^ The primary indications for liver transplant for children with Alagille syndrome are often related to the complications of chronic cholestasis (72%), such as intractable pruritus, growth failure, xanthomas, metabolic bone disease, and/or fat-soluble vitamin deficiency, or portal hypertension (30%).^13^ It is critical to assess cardiac function prior to liver transplant for children and adults with Alagille syndrome (eg, echocardiogram) to assess for any cardiac involvement, as this can affect both perioperative management, as well as post-transplant management. Head and abdominal imaging should also be completed to identify any vascular anomalies. Patients generally do well after liver transplant with reported patient survival of 5, 10, and 20 years of 92%, 91%, and 88% under the GALA study.^13^ Liver transplant is typically considered when patients with PFIC have end-stage liver disease, HCC, or refractory pruritus. For those with severe FIC1 and BSEP deficiency, native liver survival beyond childhood is unlikely with only 50% of patients surviving with a native liver at the 10-year mark.^1^

Liver transplant can be a definitive treatment modality for those with BSEP and MDR3 mutation. However, for those with FIC1 disease, liver transplant can lead to further complications such as pancreatitis, allograft steatosis, and diarrhea related to extrahepatic expression of *ATP8B1*.^40^ In those with PFIC1 without cirrhosis, nontransplant surgical options should be strongly considered before a liver transplant. There is one case report describing the use of internal biliary diversion at the time of liver transplant for a child with PFIC1 to alleviate these post-transplant symptoms.^80^

## CONCLUSIONS

Molecular testing and advancements have aided in the diagnosis of both Alagille syndrome and PFIC. Unfortunately, for those who are affected, both can lead to impaired quality of life due to its hepatic and extrahepatic involvement. While liver transplant has been performed and can be successful for both, new medical therapy such as ileal bile acid transport inhibitors may help alleviate many of the symptoms and potentially avoid liver transplant. The long-term use of ileal bile acid transport inhibitors and their effect on quality of life, liver disease progression, and reversal of pruritus and cholestasis will be anxiously awaited. Given both of these diseases’ complexity and multi-organ involvement, it often requires multidisciplinary team management to care for children and adults with Alagille syndrome or PFIC.
